# Trim69 regulates zebrafish brain development by ap-1 pathway

**DOI:** 10.1038/srep24034

**Published:** 2016-04-06

**Authors:** Ruiqin Han, Renxian Wang, Qing Zhao, Yongqing Han, Shudong Zong, Shiying Miao, Wei Song, Linfang Wang

**Affiliations:** 1 National Laboratory of Medical Molecular Biology, Institute of Basic Medical Sciences, Chinese Academy of Medical Sciences and Peking Union Medical College, Beijing, 100005, China; 2 National Health and Family Planning Commission of the People’s Republic of China, WHO Collaboration Center of Human Reproduction, Beijing 100081, China

## Abstract

Proteins belonging to the TRIM family have been implicated in a variety of cellular processes such as apoptosis, differentiation, neurogenesis, muscular physiology and innate immune responses. *Trim69*, previously identified as a novel gene cloned from a human testis cDNA library, has a homologous gene in zebrafish and this study focused on investigating the function of *trim69* in zebrafish neurogenesis. *Trim69* was found to be expressed in zebrafish embryo brain at the early stages. Knockdown of *trim69* led to deformed brain development, obvious signs of apoptosis present in the head, and decreased expression of neuronal differentiation and stem cell markers. This phenotype was rescued upon co-injection of human mRNA together along with the *trim69* knockdown. Results of this study also showed an interaction between TRIM69 and c-Jun in human cells, and upon TRIM69 knock down c-Jun expression subsequently increased, whereas the over-expression of TRIM69 led to the down-regulation of c-Jun. Additionally, knockdown both *c-Jun* and *trim69* can rescue the deformed brain, evident cellular apoptosis in the head and decreased expression of neuronal differentiation and stem cell markers. Overall, our results support a role for *trim69* in the development of the zebrafish brain through *ap-1* pathway.

The process of vertebrate neurogenesis involves in a variety of cellular processes; including, but not limited to, proliferation, apoptosis and differentiation. A delicate balance among proliferation, apoptosis and differentiation is vital to the process of neurogenesis during all stages of vertebrate development. Among of them, the processes of apoptosis and differentiation are especially critical in vertebrate neurogenesis, as they act as a determinant in the fate of neuronal precursor cells^1^.

The AP-1 signaling pathway plays a role in a variety of cellular processes including differentiation, proliferation, and apoptosis^2^. The activator protein 1 (AP-1) is a hetero dimeric transcriptional factor, comprised of proteins belonging to the c-Fos, c-Jun, ATF and JDP families. Its primary role is the regulation of gene expression in response to a variety of stimuli; including growth factors, cytokines, stress, and viral and bacterial infections. AP-1 is often thought of as a general nuclear decision-maker; such that it typically determines the fate of a cell in response to extracellular stimuli^3^. c-Jun, a key gene of AP-1 pathway, was ubiquitously expressed in rat during embryogenesis, and starting with neurogenesis^4^. Its upstream kinases (JNK2 alpha1, MKK4 and MEKK4) were also highly expressed in neuroepithelium, too^5^,^6^. In the forebrain and hindbrain, neuroblasts with high levels of nuclear p-c-Jun migrating out of the neuroepithelial layer to form the marginal zone^7^. And in the developing hindbrain, the expression of c-Jun was regulated by the transcription factor MafB, but not KROX20. Meanwhile, Jun and MafB can also form a heterodimer to regulate HOxB3. This mechanism may play a role in the patterning of hindbrain^7^.

The TRIM protein family (The tripartite motif protein family, TRIM), also called the RBCC family, consists of proteins containing a tripartite motif composed of a RING domain, one or two B-box domains and a Coiled-coil region^8^. Proteins belonging to this family have been implicated in a variety of cellular processes; including apoptosis, cell cycle regulation, neurogenesis, muscular physiology and innate immune responses^9^,^10^. Previously identified in our lab, TRIM69, a novel gene cloned from a human testis cDNA library, has a homologous gene in vertebrates. Much like other TRIM protein family members, TRIM69 also contains a classic RBCC domain. Some evidence supports a role for TRIM proteins in neurogenesis, however, whether TRIM69 has a similar function remains unclear. Due to the characteristic transparent embryos, cheap cost, wide-spread availability, ease of both *in vitro* fertilization and *in vitro* development, and their simple manipulation; zebrafish are becoming an ideal model organism for research studies^11^. In this study, we focused on investigating the role of *trim69* in zebrafish neurogenesis and the molecular mechanism through which it carries out its function.

## Results

### *Trim69* was expressed in zebrafish embryos brain at various early developmental stages

**Figure 1:**
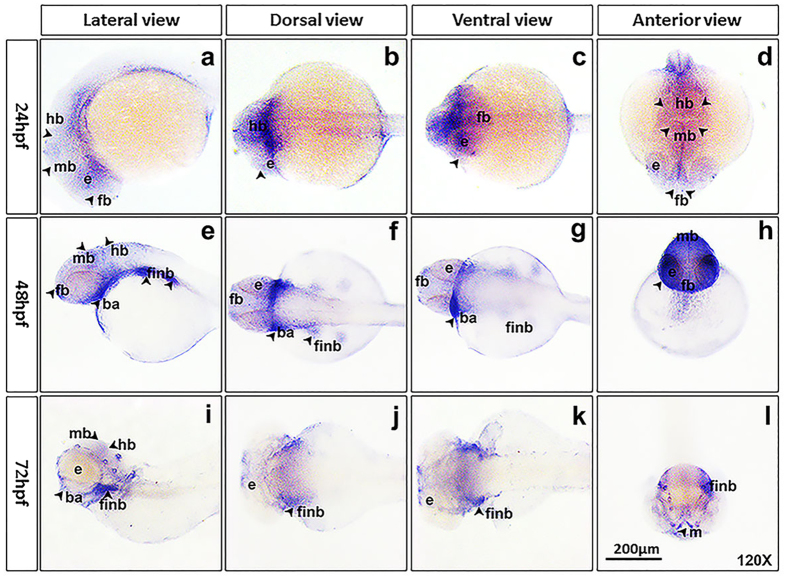
*Trim69* was expressed in zebrafish embryos’ brain. (**a–d**) trim69 was expressed in zebrafish embryo brain at 24hpf; (**e–h**) trim69 was expressed in zebrafish embryo brain at 48hpf; (**i–l**) trim69 was expressed in zebrafish embryo brain at 72hpf; e: eye; fb: forebrain; mb: midbrain; hb: hindbrain; finb: fin bud; ba: brachial arch; scale bar: 200 μm.

Whole mount *in situ* hybridization was done in order to examine the expression of *trim69* in zebrafish embryos during different stages of development. It was found that *trim69* was ubiquitously expressed in zebrafish embryos’ brain of 24hpf, 48hpf, and 72hpf. And it was restrictedly expressed in the eye, forebrain, midbrain, and hindbrain at stage of 24hpf, and was also expressed in fin bud and brachial arch at stages of 48hpf and 72hpf (Fig. 1).

### *Trim69* knock down causes defect phenotype in zebrafish embryo brain

**Figure 2:**
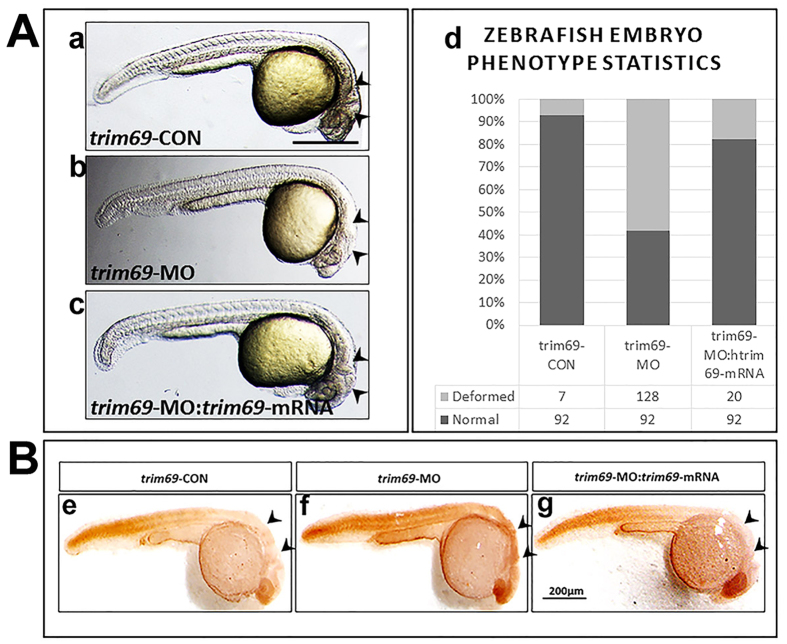
*Trim69* knockdown induces deformed brain and apoptosis. (**A**) The zebrafish embryo phenotype after *trim69* knock-down. *trim69*-CON(a): control group; *trim69*-MO(b): *trim69* knocking down; *trim69*-MO:*trim69*-mRNA(c): co-injection with *trim69*-MO and human *trim69* mRNA; black arrow indicates mid-hind brain boundary(MHB); scale bar: 200 μm; (d) Statistical analysis of the zebrafish embryo phenotype. Normal: the phenotype of the zebrafish embryo is normal; deformed: the phenotype of the zebrafish embryo phenotype is abnormal. (**B**) The examined apoptosis of zebrafish embryos by TUNEL assay. *trim69*-CON(e): control group; *trim69*-MO(f): *trim69* knocking down; *trim69*-MO:*trim69*-mRNA(g): co-injection with *trim69*-MO and human *trim69* mRNA; black arrow indicates apoptotic cells; scale bar: 200 μm.

The expression of *trim69* in very early developmental stages of zebrafish embryo brain suggests a role for this protein in brain development. In order to determine its role in zebrafish development, we examined the loss of function phenotype utilizing *trim69*-MO antisense oligonucleotides. Each embryo was injected with 5 ng *trim69*-MO, incubated in embryo medium for 24 hrs at 28.5 °C and monitored using a stereo dissection microscope. Results indicated that the loss of *trim69* in embryogenesis led to significant defects in brain development. Specifically, the mid-hind brain boundary (MHB) was either too subtle to identify or absent (Fig. 2A(b) and Fig. s1). Any embryo found to have a brain defect was then classified as deformed and the number of deformed and normal embryos was counted for both the *trim69*-MO and the control group. The ratio of deformed to normal embryos was found to be significantly higher in the *trim69*-MO group as compared to the control group (Fig. 2A(d)). These results suggest a role for *trim69* in the formation of the mid-hind brain boundary (MHB) in zebrafish embryos.

### *Trim69* knock down induces apoptosis in zebrafish embryo brain

The above results showed that the loss of *trim69* led to obvious defects in the development of the brain in zebrafish embryos; however, the underlying mechanism still remained unclear. We hypothesized that the observed phenotype was due to an increase in the biological process of cell death and performed a TUNEL assay to examine the number of apoptotic cells in both the *trim69*-MO and control groups. As expected, the *trim69*-MO group had a greater number of apoptotic cells in comparison to control group (Fig. 2B(f)). We further detected the expression of apoptotic related genes (bax and cleaved caspase3) by qPCR. We found that the results of qPCR were in accordance with the TUNEL assay (Fig. s2).

### Human *trim69* mRNA can rescue the defective phenotype caused by zebrafish *trim69*-MO

The relative conservation of the amino acid sequence, including similar motifs and domains, of TRIM69 between human and zebrafish suggests that these proteins have similar functions. Thus, it was hypothesized that human *trim69* mRNA could rescue the defective phenotype caused by the injection of zebrafish *trim69*-MO in zebrafish embryos. The human *trim69* mRNA was co-injected with zebrafish *trim69*-MO, incubated for 24hpf and then checked by a stereo microscopy. As expected, the co-injection of human *trim69* mRNA with zebrafish *trim69*-MO rescued the defects observed with the injection of *trim69*-MO alone (Fig. 2A(c,d) and Fig. s1).

### Human *trim69* mRNA can rescue the apoptosis induced by zebrafish *trim69*-MO

We further investigated if rescuing the defective phenotype through the co-injection of zebrafish *trim69*-MO with human *trim69* mRNA also rescued the increase in apoptotic cell death observed after the injection of *trim69*-MO alone. TUNEL assay analyses indicated that the human TRIM69 mRNA can also rescue the observed apoptosis induced by zebrafish *trim69*-MO (Fig. 2B(g)).qPCR assay analyses further indicated that the human *trim69* mRNA can rescue the apoptosis induced by zebrafish *trim69*-MO (Fig. s2).

### *Trim69* knock down down-regulated the expression of neuronal differentiation markers

**Figure 3:**
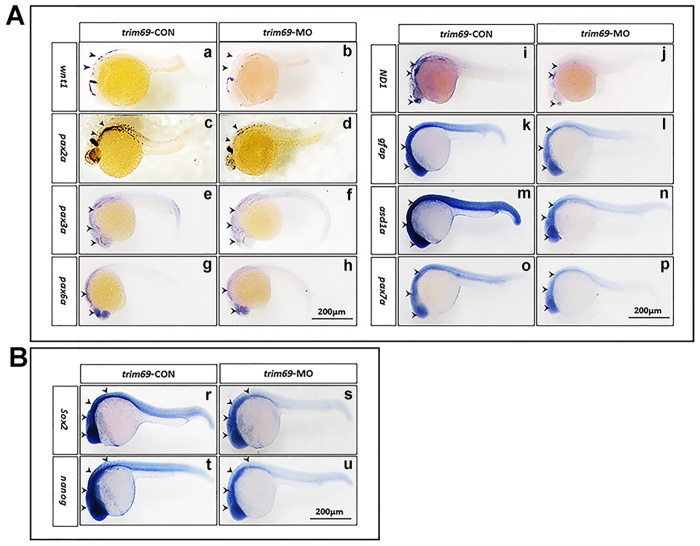
*Trim69* knockdown affects zebrafish embryo neurogenesis. (**A**) The expression of neuronal differentiation markers was decreased in response to *trim69* knockdown. *trim69*-CON: control group; *trim69*-MO: *trim69* knocking down; black arrow indicates positive signals; scale bar: 200 μm; (**B**) The expression of stem cell markers was decreased in response to *trim69* knockdown. *trim69*-CON: control group; *trim69*-MO: *trim69* knocking down; black arrow indicates positive signals; scale bar: 200 μm.

To the neurogenesis, apoptosis is essential but not limited, differentiation is also a vital process. We synthesized the probes of neuronal differentiation markers and conducted the whole mount *in situ* hybridization found that the expression of *wnt1, pax2a, pax3a, pax6a, pax7a, neuroD1, gfap, and ascl1a* were decreased in response to trim69 knockdown (Fig. 3A). To further ascertain the above results, we detected the expression of *wnt1, pax2a, pax3a, pax6a, pax7a, neuroD1, gfap, and ascl1a* by qPCR; it was found that these markers’ expression were significantly decreased after *trim69* knockdown, and the co-injection with human *trim69* mRNA can rescue their expression level (Fig. s3). These findings were in accordance with the whole mount *in situ* hybridization.

### *Trim69* knock down decreased the expression of stem cell makers

We also checked the expression of stem cell makers by whole mount *in situ* hybridization. It was found that the expression level for stem cell markers, *sox2* and *nanog*, were decreased obviously compared to control group (Fig. 3B). We performed the qPCR to further test the results for whole mount *in situ* hybridization. We found that expression of *sox2* and *nanog* was significantly decreased by *trim69* knocking down, and co-injection with human *trim69* mRNA can rescue their expression level (Fig. s3). These findings were in accordance with the whole mount *in situ* hybridization.

### *Trim69* knock down decreased the expression of brain region specific makers

We performed the *in situ* hybridization to determine the expression of *otx2* (marker of forebrain and MHB), *eng2a* (marker for MHB and hindbrain), *egr2b* (marker for MHB and hindbrain), and *fgf8a* (marker for forebrain and MHB) after loss of *trim69.* We found that the expression of *otx2, eng2a, egr2b,* and *fgf8a* was reduced after loss of *trim69* (Fig. s4).

### *TRIM69* specifically inhibits *AP-1* activation

**Figure 4:**
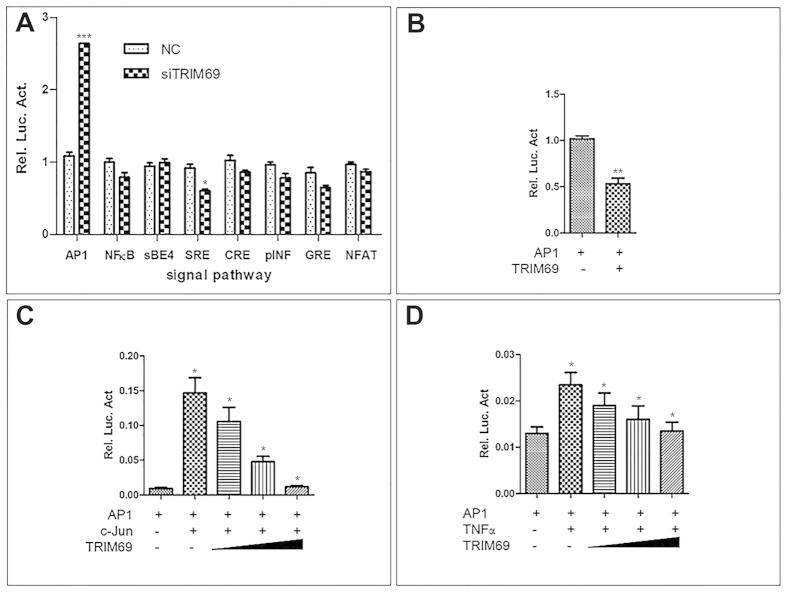
TRIM69 specifically inhibits AP-1 activation. (**A**) HEK 293T cells were co-transfected with an AP-1-driven luciferase reporter and the indicated siRNA of TRIM69. Relative luciferase activity was measured and statistically analyzed by unpaired t-test, p < 0.01; (**B**) HEK 293T cells were co-transfected with signal-pathway-driven luciferase reporter plasmids and Flag-TRIM69. Relative luciferase activity was measured and statistically analyzed by unpaired t-test, p < 0.01; (**C**) HEK 293T cells were transiently transfected with different concentrations of Flag-TRIM69 (0.2, 0.4, and 0.8), AP-1-driven luciferase reporter and c-Jun. Relative luciferase activity was measured and statistically analyzed by unpaired t-test, p < 0.05; (**D**) HEK 293T cells were transiently transfected with different concentrations of Flag-TRIM69 (0.2, 0.4, and 0.8) and AP-1-driven luciferase reporter. Cells were treated with 10 ng/ml TNFα for 8–12 h. Relative luciferase activity was measured and statistically analyzed by unpaired t-test, p < 0.05.

To further investigate the mechanism by which *trim69* affects zebrafish mid-hindbrain boundary development, we first tested the role of *trim69* in the signaling pathway process. A reporter gene analyses in HEK293T cells was done using luciferase assays. Our results indicated that the overexpression of *TRIM69* can inhibit the AP-1 transcriptional activity. Notably, this inhibitory effect on AP-1 activation appeared to be specific due to the lack of an effect observed for other pathways (Fig. 4B). As expected, knockdown of endogenous *TRIM69* led to an increased activation of the *AP1* signaling pathway (Fig. 4A). Next, we found that *TRIM69* could also inhibit the potentiation of *AP-1* reporter gene activity induced by co-transfecting *c-Jun* in a dose-dependent manner (Fig. 4C,D). Overall, these results suggest that *TRIM69* specifically inhibits the *AP-1* signaling pathway.

### TRIM69 negatively regulates the expression of c-Jun

**Figure 5:**
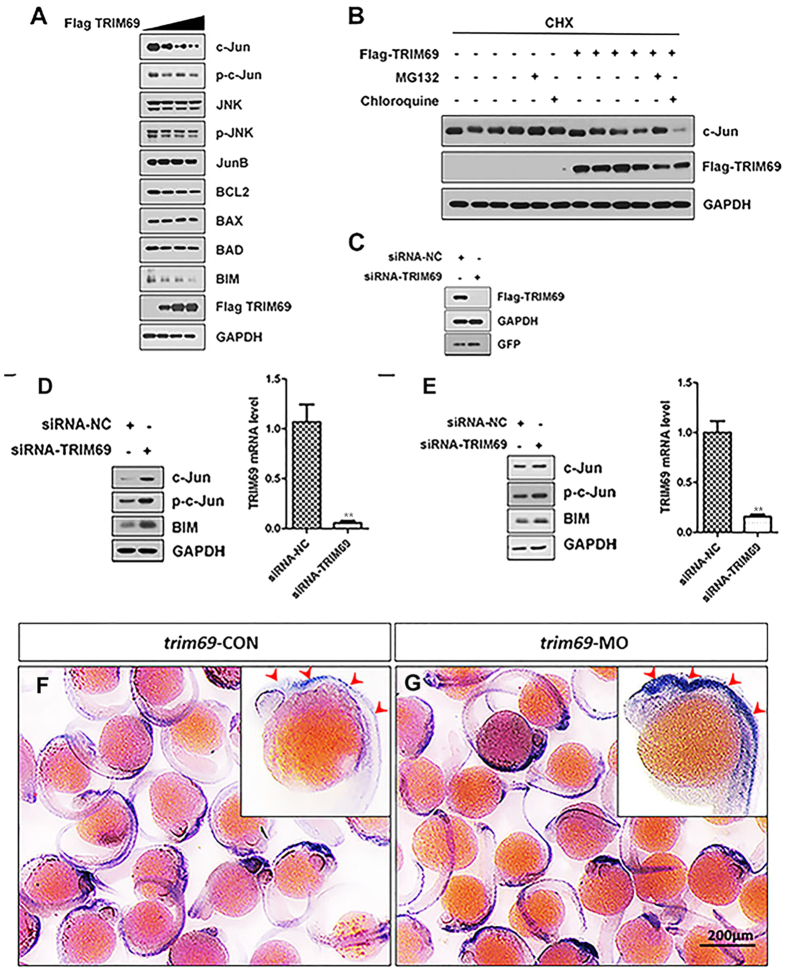
TRIM69 negatively regulates the expression of c-Jun. (**A**) HEK 293T cells were transfected with Flag-TRIM69 or a control expression vector. The cell lysates were analyzed by immunoblotting with the indicated antibodies. GAPDH was used as a loading control. (**B**) HEK 293T cells were transfected with Flag-TRIM69 or a control expression vector. At 36 h post-transfection, the cells were treated with cycloheximide (200 μg/ml, CHX) and MG132 (20 μM) or chloroquine (100 μM) for 2 h. (**C**) HEK 293T cells were co-transfected with Flag-TRIM69 along with siRNA-NC or siRNA-TRIM69. The cell lysates were analyzed by immunoblotting with the indicated antibodies. GAPDH was used as a loading control. (**D,E**) SH-SY5Y and HEK 293T cells were transfected with siRNA-NC or siRNA-TRIM69. The cell lysates were analyzed by immunoblotting with the indicated antibodies. GAPDH was used as a loading control. Simultaneously, the relative RNA level of TRIM69 was determined by qPCR. Data were measured in triplicate and statistically analyzed by unpaired t-test, p < 0.01; values and bars represent the mean and standard deviation, respectively; (**F,G**) *trim69* knockdown decreased the expression of *c-Jun* in zebrafish. *trim69*-CON: control group; *trim69*-MO: *trim69* knocking down; scale bar: 200 μm.

Based on the above results, it was hypothesized that TRIM69 may inhibit the AP-1 signaling pathway by regulating an AP-1 transcription factor. To investigate this hypothesis, the expression levels of the major known AP-1 transcription factors and their downstream apoptotic related proteins were examined both before and after TRIM69 overexpression. Results indicated that the transient transfection of TRIM69 decreased the cellular level of c-Jun, p-c-Jun and BIM in a dose-dependent manner (Fig. 5A). Notably; the over-expression of TRIM69 not only down regulated the expression of endogenous c-Jun but also increased its rate of degradation; as measured by a cycloheximide-chase assay (Fig. 5B). The TRIM69-dependent loss of c-Jun was also shown to be blocked after the addition of the proteasome inhibitor MG132 but not after the addition of the lysosome inhibitor chloroquine (Fig. 5B). To support the conclusions based on the results of over-expressing TRIM69; the effects of knocking-down endogenous TRIM69 on protein expression was also determined. The specificity and efficacy of our strategy; which was to use SMARTpool siRNA targeting of TRIM69, was determined by cotransfecting Flag-TRIM69 along with the SMARTpool siRNA in HEK293T cells (Fig. 5C).Immunoblot analysis and reverse transcription-PCR (RT-PCR) confirmed both the specificity and efficacy of our SMARTpool siRNA targeting TRIM69. We validated our strategy in this manner because the available TRIM69 antibodies, both privately made and commercially available, do not work well. The expression levels of c-Jun, p-c-Jun and BIM were shown to increase when TRIM69 was silenced in both SH-SY5Y and HEK293T cells (Fig. 5D,E). We also determined the expression of c-Jun response to *trim69* knockdown in zebrafish and found that its results was consistent with *in vitro* (Fig. 5F,G).

### TRIM69 Interacts with c-Jun

**Figure 6:**
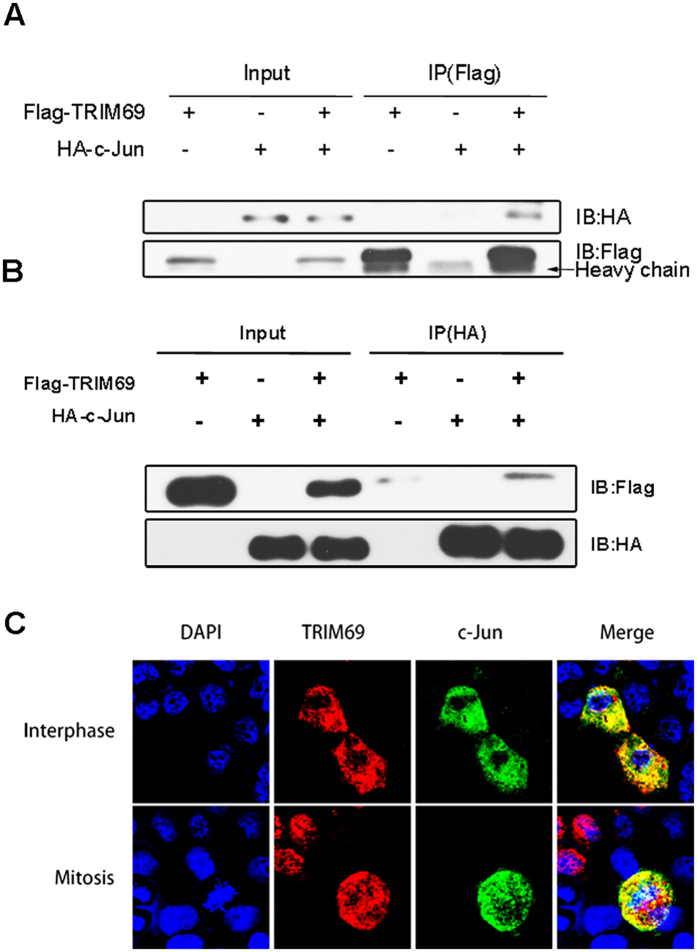
TRIM69 interacts with c-Jun. (**A,B**) HEK-293T cells were transfected with Flag-TRIM69 and HA-c-Jun. After immunoprecipitation with anti-Flag beads, HA-c-Jun was detected by immunoblotting using an anti-HA antibody; after immunoprecipitation with anti-HA beads, Flag-TRIM69 was detected by immunoblotting using an anti-Flag antibody. (**C**) HeLa cells were co-transfected with Flag-TRIM69 and HA-c-Jun. Subcellular localization of TRIM69 and c-Jun was analyzed by immuno-fluorescence (IF) with anti-Flag and anti-HA antibodies. Nuclei were stained with Hoechst33258.

To determine whether TRIM69 interacts with c-Jun; we co-transfected HEK293T cells with Flag-tagged TRIM69 and HA-tagged c-Jun or a Flag-control plasmid and performed immunoprecipitation analysis. C-Jun was consistently detected in immunoblots of Flag-TRIM69 immunoprecipitates but not in Flag-control immunoprecipitates (Fig. 6A). The interaction between HA-c-Jun and Flag-TRIM69 was further confirmed in a reverse immunoprecipitation experiment in which Flag-TRIM69 was detected in HA-c-Jun immunoprecipitates (Fig. 6B). The immunostaining patterns of TRIM69 and c-Jun in HeLa cells co-expressing Flag-TRIM69 and HA-c-Jun also suggest that these two proteins were co-localized (Fig. 6C).

### *C-Jun-MO* can rescue the defects phenotype caused by *trim69*-MO

**Figure 7:**
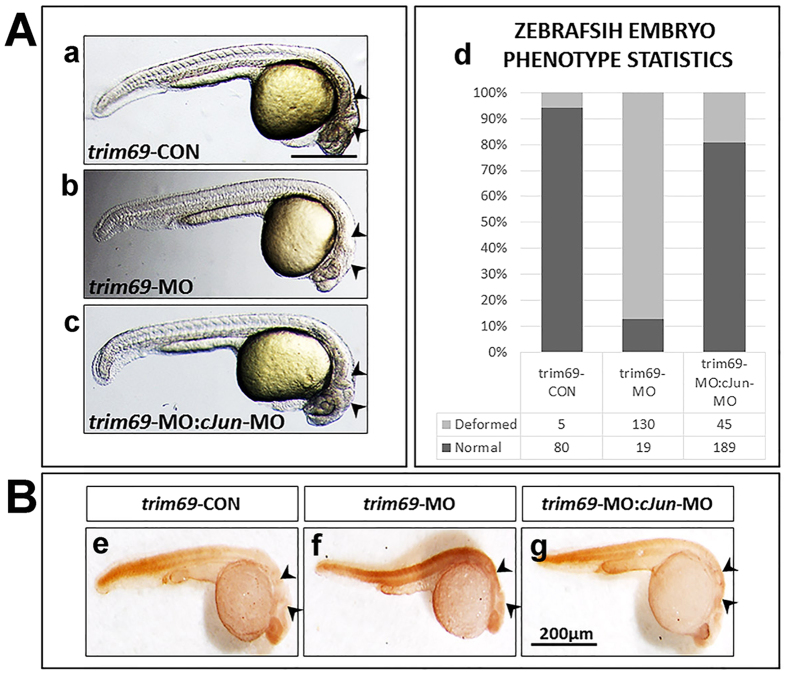
Loss of *c-Jun* rescued the deformed brain and apoptosis induced by *trim69* knockdown. (**A**) Loss of *c-Jun* rescued the deformed brain induced by *trim69* knockdown. *trim69*-CON(a): control group; *trim69*-MO(b): *trim69* knocking down; *trim69*-MO:*c-Jun-*MO(c): co-injection with *trim69*-MO and *c-Jun*-MO; black arrow indicates mid-hind brain boundary(MHB); scale bar: 200 μm; (d) Statistical analysis of the zebrafish embryo phenotype. Normal: the phenotype of the zebrafish embryo is normal; deformed: the phenotype of the zebrafish embryo phenotype is abnormal. (**B**) The examined apoptosis of zebrafish embryos by TUNEL assay. *trim69*-CON(e): control group; *trim69*-MO(f): *trim69* knocking down; *trim69*-MO:*cJun-*MO(g): co-injection with *trim69*-MO and *c-Jun*-MO; black arrow indicates apoptotic cells; scale bar: 200 μm.

As previously mentioned, c-Jun is a member of the AP-1 pathway family and the AP-1 pathway has been shown to play a role in apoptosis and differentiation. Taking into consideration the results discussed above, we designed an experiment to examine the effect of knocking down both *c-Jun* and *trim69* in zebrafish embryos. *Trim69-MO* and *c-Jun*-MO were co-injected into zebrafish embryos, incubated at the 24hpf stage and then checked by a stereo microscopy. Interestingly, the deformed phenotype observed was shown to be partially rescued by co-injecting with *c-Jun-*MO (Fig. 7A and Fig. s1).

### *C-Jun-MO* can rescue the cell apoptosis induced by *trim69*-MO

Considering that the AP-1 pathway has been shown to be involved in the apoptotic pathway; and c-Jun is one member of the AP-1 pathway that appeared to partially rescue the deformed phenotype induced by the *trim69*-MO, we determined if this also led to a reduction in apoptosis noted for *trim69*-MO alone. The *c-Jun-*MO and *trim69*-MO were co-injected in zebrafish embryos and incubated at of the 24hpf stage and then observed by microscopy. As expected, the co-injection *c-Jun-*MO was shown to rescue the apoptosis induced by *trim69*-MO alone (Fig. 7B). To further confirm the above results of the TUNEL assay, the expression of *bax* and cleaved *caspase3* was determined by qPCR. It was found that the expression of *bax* and *cleaved caspase3* were significantly decreased after *trim69* knockdown and they can be rescued by co-injection with *c-Jun*-MO (Fig. s2). This finding was in accordance with the TUNEL assay.

### *C-Jun-MO* can rescue the neuronal differentiation markers’ expression

**Figure 8:**
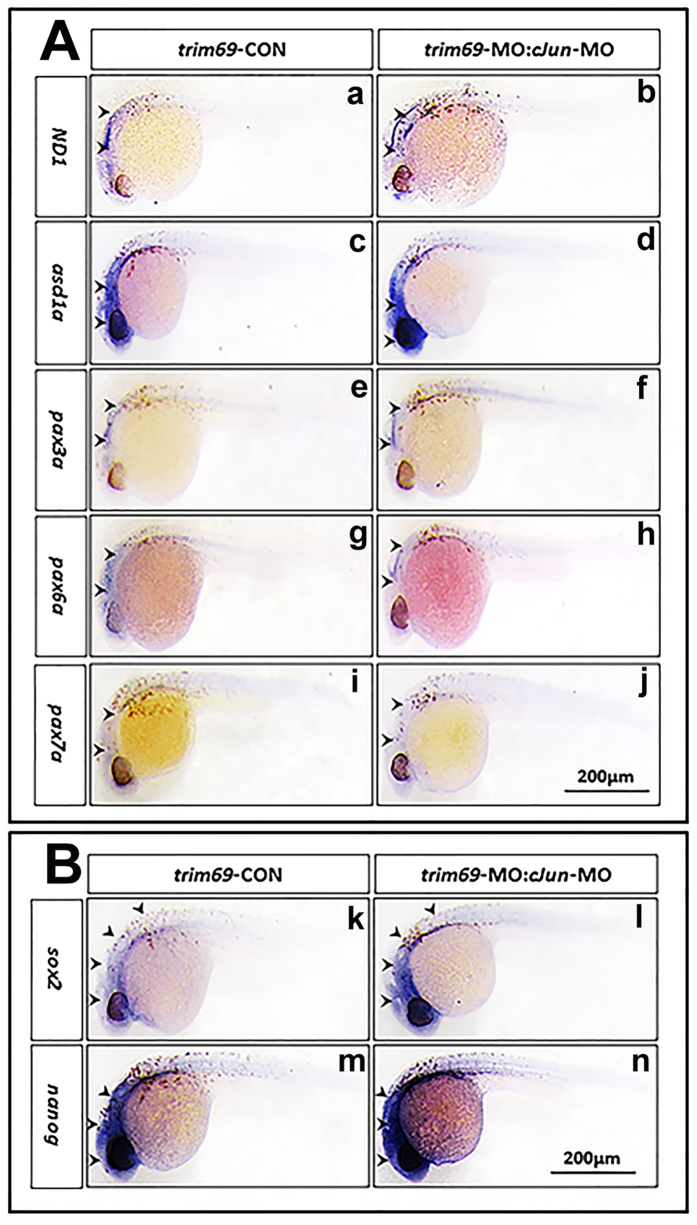
Loss of *c-Jun* rescued the zebrafish embryo neurogenesis. (**A**) Loss of *c-Jun* rescued the expression of neuronal differentiation markers. *trim69*-MO(f): *trim69* knocking down; *trim69*-MO:*cJun-*MO(g): co-injection with *trim69*-MO and *c-Jun*-MO; black arrow indicates positive signals; scale bar: 200 μm; (**B**) Loss of *c-Jun* rescued the expression of stem cell markers. *trim69*-MO(f): *trim69* knocking down; *trim69*-MO:*cJun-*MO(g): co-injection with *trim69*-MO and *c-Jun*-MO; black arrow indicates positive signals; scale bar: 200 μm.

Above results showed that co-injection *c-Jun*-MO can rescue the defects phenotype and apoptosis induced by *trim69* knockdown, but whether can rescue the expression of neuronal differentiation markers is not certain. We determined the expression of neuronal differentiation markers’ expression by *in situ* hybridization, it was found that co-injection *c-Jun*-MO can definitely rescued the expression level of *wnt1, pax2a, pax3a, pax6a, pax7a, gfap, and ascl1a* (Fig. 8A). qPCR also showed that the expression for neuronal differentiation markers can be rescued by co-injection with *c-Jun*-MO. This result was in accordance with the *in situ* hybridization (Fig. s3).

### *C-Jun-MO* can rescue the stem cell markers’ expression

We also determined the expression of stem cell markers’ expression by whole mount *in situ* hybridization, and as we had expected, the expression of *sox2* and *nanog* was rescued by co-injection *c-Jun-*MO compared to *trim69-*MO alone (Fig. 8B). qPCR also showed that the expression for stem cell markers can be rescued by co-injection with *c-Jun*-MO and was in accordance with the *in situ* hybridization (Fig. s3).

## Discussion

TRIM proteins are a family of intracellular proteins sharing the RBCC motif or the N-terminal tripartite. Thirty-seven TRIM proteins have been identified in humans and play a variety of roles in animal development and cell growth^12^,^13^. TRIM9 has been reported to be specifically expressed in the embryonic and adult nervous system. During embryogenesis, it has been reported to have a high expression in the developing neocortex, the dorsal thalamus, the midbrain, the basal area of the hindbrain and the spinal cord. In the adult brain, TRIM9 is expressed in the Purkinje cells of the cerebellum, in the hippocampus and in the cortex^14^,^15^. TRIM9 and TRIM69 are both members of the TRIM protein family and their similar protein structures suggest that they may serve similar functions during animal development. Here we show that *trim69* is restrictedly expressed in zebrafish embryo brain at 24hpf, 48hpf, 72hpf, this expression pattern is similar to TRIM9. This data supports a role for *trim69* in the brain development of zebrafish.

Previously published results support a role for TRIM proteins in neuronal development. The TRIM-NHL protein (TRIM32) was shown to activate a microRNA and prevent self-renewal in mouse neuronal progenitors^16^,^17^,^18^. TRIM proteins have also been shown to promote neuronal differentiation through retinoic acid receptor-mediated transcription^18^. TRIM-2/3 (L-TRIM) has been shown to be up-regulated during *in vitro* experiments studying neurite outgrowth of central neurons. In adult animals, L-Trim mRNA is ubiquitously expressed at low levels in the central nervous system as well as in peripheral tissues. Levels of L-Trim mRNA were also shown to increase during postnatal brain development and during *in vitro* and *in vivo* neuronal regeneration of the central nervous system. *In vitro* double-stranded RNA knock-down of L-Trim mRNA resulted in a N70% inhibition of neurite outgrowth^19^,^20^. In the nematode Caenorhabditis elegans, the let-7 microRNA (miRNA) controls the timing of key developmental events and terminal differentiation in part by directly regulating lin-41 (CLIN41/TRIM71). C. elegans lin-41 mutants show precocious cell cycle exit and terminal differentiation of epidermal skin cells. Lin-41 orthologues are found in more complex organisms such as mice and humans, however, their functions remain unknown. Mlin41 mouse mutants showed an obvious neural tube closure defect during development, resulting in embryonic lethality. As was similarly noted for lin-41 in C. elegans, Mlin41 also appears to be regulated by the let-7 and mir-125 miRNAs. Considering that Mlin41 is required for neural tube closer and survival, human lin-41 (HLIN41/TRIM71) is likely important in human development and disease^21^. In this study, using zebrafish as a model, we investigated the role of *trim69* in the development of the brain of zebrafish embryo’s using a variety of techniques. Knock-down of *trim69* was shown to result in the disappearance of the mid-hind brain boundary. This supports the hypothesis that *trim69* may play a key role in the formation of the zebrafish mind-hind brain boundary.

Both the mRNA and protein expression of Trim17, one of the members of the TRIM/RBCC protein family, was found to be increased in several *in vitro* models of transcription-dependent neuronal apoptosis. Additionally, the Trim17 protein is expressed *in vivo* in apoptotic neurons as they naturally die during post-natal cerebellar development. The overexpression of an active Trim17 in primary CGN was shown to be sufficient to induce the intrinsic pathway of apoptosis in survival conditions. Furthermore, the knock-down of endogenous Trim17 and the overexpression of dominant-negative mutants of Trim17 were shown to block trophic factor withdrawal-induced apoptosis both in CGN and in sympathetic neurons^22^. The disappearance of the mi-hind brain after knockdown of *trim69* that we noted after knockdown of *trim69* led us to further investigate the mechanism behind this phenotype. As it was a tissue structure defect, we hypothesized that it may be a result of an effect on the apoptotic pathway. A TUNEL assay was performed to estimate whether the phenotype was related to an effect on apoptotic pathway. As expected, a greater number of apoptotic cells were present in the brain of zebrafish embryos from the *trim69*-MO group in comparison to control group. Thus, supporting the hypothesis that the abnormal phenotype observed upon depletion of *trim69* is mediated by the apoptotic pathway.

Zebrafish TRIM69 has a homologous gene in humans; the ability of human *trim69* mRNA to rescue the phenotype induced by zebrafish *trim69*-MO suggests that the two proteins have similar functions. This implies that molecular studies of *trim69* in human cells lines can be applicable in determining the role of *trim69* in zebrafish neurogenesis.

In order to investigate the molecular mechanism of *trim69* and the role it plays in zebrafish brain development, we examined the effect that either overexpression or depletion of *trim69* has on various signaling pathways. Our results showed that the overexpression of *TRIM69* alone can inhibit *AP-1* transcriptional activity. Notably, the inhibitory effect on *AP-1* activation by *TRIM69* appeared to be specific. Meanwhile, the depletion of endogenous *TRIM69* was shown to markedly activate the AP1 signaling pathway. Additionally, we found that *TRIM69* was able to inhibit AP-1 reporter gene activity induced by co-transfected c-Jun in a dose-dependent manner. Our data also showed that *TRIM69* was capable of inhibiting tumor necrosis factor (TNF) induced *AP-1* transcriptional activity in a dose dependent manner. Overall, these results suggest that *TRIM69* specifically inhibits the *AP-1* signaling pathway. The *AP-1* signaling pathway plays a role in a variety of cellular processes including differentiation, proliferation and apoptosis^3^. The activator protein 1 (AP-1) is a heterodimeric transcription factor comprised of proteins belonging to the *c-Fos, c-Jun, ATF and JDP* families. It regulates gene expression in response to a variety of stimuli; including stress, growth factors, cytokines, and both viral and bacterial infections. *AP-1* is often regarded as a general, nuclear decision-maker that determines life or death cell fates in response to extracellular stimuli^2^.

C-Jun is a protein that is encoded by the *JUN* gene in humans. It is highly similar to the viral protein and directly interacts with specific target DNA sequences in order to regulate gene expression^23^. Both c-Jun and c-Fos constitute the AP-1 early transcription factor response. It is activated through two different mechanisms; one is the double phosphorylation by the JNK pathway and the other is a phosphorylation-independent event^24^. The product of c-Jun, Jun, regulates its transcription^25^. Our experimental results indicate that the endogenous protein level of c-Jun, p-c-Jun and BIM are all down-regulated in a dose-dependent manner and increase the degradation of c-Jun after transient transfection of vectors encoding TRIM69. In turn, the expressional levels of c-Jun, p-c-Jun and BIM were increased upon TRIM69 depletion in both SH-SY5Y and HEK293T cells; indicating that TRIM69 may inhibit c-Jun’s transcriptional activation. The activation of Jun is induced by the binding of AP-1 to the high affinity AP-1 binding site in the promoter region Jun. This mechanism is positively auto-regulated by activating its own transcription with the aim at prolonging the signals from the extracellular stimuli. This type of mechanism possesses important biological significance in cancer^26^. Additionally, c-Jun activity can also be regulated by the ERK pathway by affecting both c-Jun transcription and stability. This leads to the activation of c-Jun and its downstream targets, including RACK1 and cyclin D1. JNK activity can be enhanced by RACK1 and c-Jun activity is subsequently regulated by activated JNK signaling. It is shown that Jun’s activity, AP-1 activity, in stress-induced apoptosis and cellular proliferation is regulated by phosphorylation of N-termini^24^. Our results show that the overexpression of TRIM69 down regulated the expression of c-Jun, p-c-Jun, BIM, and the JNK. We also showed that TRIM69 co-localized with c-Jun and that they appear to interact. This data indicates that TRIM69 may compete with c-Jun for the AP-1 binding site in the promoter region to prevent c-Jun transcriptional activation.

Overall our results indicate that TRIM69 can negatively regulate the expression of c-Jun and interact with it in human cell lines; whether or not these results can be recapitulated in zebrafish remains to be determined. Co-injecting *c-Jun-MO* and *trim69-MO* in zebrafish embryos was shown to rescue the abnormal phenotype, the increase in cellular apoptosis, and the expression of neuronal differentiation and stem cell markers induced by *trim69*-MO alone. These results indicate that the results obtained using human cell lines is consistent with the results obtained using zebrafish. Considering that TRIM69 was shown to interact with c-Jun and negatively regulate its expression and that depleting c-Jun resulted in the rescue of both the deformed phenotype, the increase in cellular apoptosis and the decreased expression of neuronal differentiation and stem cell markers; we can infer that the deformed phenotype, being the disappearance of the MHB, is induced by c-Jun mediated cell apoptosis and differentiation.

## Materials and Methods

### Zebrafish and embryos

Zebrafish were maintained in a recirculating aquaculture system with a 14 h light and 10 h dark cycle at 27–28 °C; Embryos were incubated in embryo medium (5 mM NaCl, 0.17 mM KCl, 0.33 mM CaCl2, and 0.33 mM MgSO4) at 28.5 °C. All developmental stages were determined according to Kimmel *et al.*^27^.

The corresponding author declares that all the methods were carried out in accordance with the approved guidelines and that all animal experiments were conducted under protocols approved by the Animal Research Committee of the Institute of Laboratory Animals, Chinese Academy of Medical Sciences and Peking Union Medical College. All surgeries were performed under sodium pentobarbital anesthesia, and all efforts were made to minimize suffering. Moreover all the technicians and researcher who take care of the animals and perform the experiments are appropriately trained by attending specific courses.

### Zebrafish total RNA extraction

Zebrafish embryos were allowed to develop to certain stages and were collected in a 1.5 mL RNAase free centrifuge tube with the addition of 1 mL TRIzol ^®^ Reagent (life technologies, USA) and homogenized using a glass power homogenizer. The following manipulations were done according to the standard protocols provided by Life Technologies.

### Zebrafish whole mount *in situ* hybridization

Different stages of zebrafish embryos were collected and fixed in 4% PFA for 24 hr to 48 hr and washed with PBS for three times, 5 min each, at RT. Subsequently, embryos were transferred to a serial graded methanol solution for dehydration and then stored in pure methanol at −20 °C or −80 °C for further use. Whole mount *in situ* hybridization of digoxigenin-labelled antisense probes were carried out according to the standard protocols^27^.

### Probe and mRNA synthesis

For the synthesis of the zebrafish *trim69* probe, a zebrafish *trim69* cDNA fragment, which contained the base-pairs from 97 bp to 983 bp of the coding region, was amplified by RT-PCR (Qiagen One Step RT-PCR kit) and cloned into the pGEM-T-EASY (Roche, USA) expression vector in order to generate pGEM-T-EASY-zf-*trim69*. Total RNA from zebrafish embryos was used as a template. The following zebrafish *trim69* specific primers were used:

Zebrafish *trim69* forward primer: 5′-GTCATGCGTGAAACTCCTGCCAAC-3′

Zebrafish *trim69* reverse primer: 5′-CACTGAGCAGAGCCTTGTGTTCGTC-3′

For the synthesis of human *trim69* mRNA, a human full length TRIM69 cDNA fragment was amplified by RT-PCR (Qiagen One Step RT-PCR kit) and cloned into the pGEM-T-EASY (Roche, USA) expression vector to generate pGEM-T-EASY-h-TRIM69. Total RNA from human cells was used as a template. The following human TRIM69 specific primers were used:

Human TRIM69 forward primer: 5′-CCCATGAAGCCCCACAGAAT-3′

Human TRIM69 reverse primer: 5′-TACCATCCCTGAGCGGGTAA-3′

The reconstructed expression vector pGEM-T-EASY-zf- *trim69* and pGEM-T-EASY-h-TRIM69 were linearized and transcribed into the zebrafish TRIM69 probe and the human TRIM69 mRNA using the DIG RNA Labeling Kit (SP6/T7) (Roche, USA).

### TUNEL assay

Different stages of zebrafish embryos were collected and fixed in 4% PFA overnight and stored at 4 °C until used. Cell death in zebrafish embryos was detected by TUNEL assay using the colorimetric TUNEL apoptosis assay kit (Beyotime, China) according to the manufactures’ instruction.

### Morpholino antisense oligonucleotides and microinjection

Morpholino antisense oligonucleotides were synthesized by Gene Tools (Philomath, USA). The MO sequences were: *trim69*-MO 5′-AGTTGGCAGGAGTTTCACGCATGAC-3′, and *c-Jun*-MO 5′-CTTGGTAGACATAGAAGGCAAAGCG-3′. For injections, MO’s were diluted in 1 × Danieu’s buffer (58 mM NaCl, 0.7 mM KCl, 0.6 mM Ca (NO3)2, 1.5 mM pH 7.6 HEPES) to 1 mg/ml. The mRNA was diluted in DEPC water to 5 ug/ml. In brief, 5 nl MO or mRNA were injected into one to two cell stage embryos using micromanipulator (Eppendorf, Germany) with a micropipette via the controllable pressure of nitrogen. The whole injection process was monitored using a stereo dissection microscope (Nikon, Japan).

### Cell Culture, plasmids and transfection

HEK-293T and SH-SY5Y cells were obtained from the Cell Resource Center of Peking Union Medical College (PUMC) and cultured in Dulbecco’s Modified Eagle’s Medium (DMEM) supplemented with 10% fetal bovine serum (FBS) in a 5% CO_2_ incubator at 37 °C. All cells were passaged every 2–3 days with 0.5 mg/ml trypsin (1:250) and 0.53 mM ethylenediaminetetraacetic acid (EDTA). Plasmids were constructed using standard cloning techniques. Expression plasmids for TRIM69 and c-Jun were cloned into a modified pcDNA6.0 vector with a Flag/HA/V5 tag or pcDNA3.1-Myc vector. The siRNA used in the silencing of TRIM69 was a SMART pool (Thermo Fisher Scientific). The sequence used for the siRNA negative control was 5′-UUCUCCGAACGUGUCACGU-3′. Plasmid transfection was performed using Lipofectamine 2000 (Invitrogen) and the siRNA transfection was performed using Lipofectamine RNAiMAX (Invitrogen) following the protocol suggested by the manufacturer.

### Luciferase assays

The pAP-1-Luc plasmid (Stratagene) was transfected into HeLa cells using lipofectamineTM 2000. Additionally, HeLa cells were co-transfected with a Renilla luciferase control vector (Promega) to monitor the transfection rates. Firefly and Renilla luciferase activities were measured using the Dual-Lucierase Reporter Assay System (Promega). Briefly, the cells were washed with PBS and lysed with a passive lysis buffer. Cell lysates were mixed with the Luciferase Assay Reagent II and the Firefly luminescence was measured using a luminometer (TD-20/20, Turner BioSystems, USA). Next, the samples were mixed with the stop reagent and the Renilla luciferase activity was measured as an internal control. The relative luciferase activity was calculated as the ratio of the Firefly luciferase activity to the Renilla luciferase activity.

### Immunoblot analysis

At the indicated times post transfection, the cells were washed with PBS, harvested, lysed in a buffer containing 50 mM Tris-HCl [pH 6.8], 10% glycerol and 2% SDS, and quantified using the BCA protein assay reagent (Pierce). The cell extracts were separated on a 10% or 12% SDS-PAGE gel and then electrophoretically transferred to a PVDF membrane (GE Healthcare) according to standard protocols. The membrane was blocked in 5% skim milk for 1 hour at room temperature and then incubated overnight with the indicated antibodies at 4 °C. The membrane was incubated with an anti-mouse or an anti-rabbit HRP-IgG (Santa Cruz) for 1 hour at room temperature. Chemiluminescence was detected using an ECL blot detection system (Engreen). The antibodies used in this study were as follows: mouse anti-c-Jun antibody from BD; mouse anti-GAPDH antibody from Santa Cruz; Rabbit anti-p-c-Jun, Rabbit anti JNK, Rabbit anti-p-JNK. Rabbit anti-JunB, Rabbit anti-BCL2, Rabbit anti-BAX, Rabbit anti-BIM, Rabbit anti-BAD antibodies from Cell Signaling Technology. Mouse anti-HA, mouse anti-Flag mouse anti-His and mouse anti-Myc antibodies were from Sigma.

### Co-immunoprecipitation

Cells were transiently transfected with pcDNA6.0-Flag-TRIM69 or pcDNA6.0-HA-c-Jun as indicated. 48 hours post-transfection, the cells were harvested and lysed in NETN buffer as previously described. Protein complexes were isolated using anti-Flag beads (Sigma, M2) or anti-HA beads (Santa Cruz). The protein complexes were solubilized in 1.2 × SDS loading buffer and analyzed by immunobloting.

### Quantitative real-time-PCR (qRT-PCR)

Total RNA from zebrafish embryos and human cell line (HEK-293T and SH SY5Y cells) was extracted using TRIzol reagent (Invitrogen) and 1 μg of isolated total RNA was converted to cDNA using the Roche Transcript First-Strand cDNA Synthesis kit (Roche). The power SYBR green master mix (Applied Biosystem) was added to the cDNA samples which were then subjected to qRT-PCR using the Step-One Real-time PCR system. The relative levels of target gene mRNA were normalized against the GAPDH housekeeping gene.

The forward primer, 5′-ACCCTGAGGAACATGCAGAAGAAA-3′, and the reverse primer, 5′-AACTCCATGGACACATGTTGCTGC-3, was used to amplify TRIM69. The forward primer 5′-TGAGTACGTCGTGGAGTCCA-3′ and the reverse primer, 5′-TAGACTCCACGACATACTCA-3′ were used to amplify GAPDH.

### Immunofluorescence

HeLa cells were transfected with Flag-TRIM69 and/or HA-c-Jun using the Lipofectamine 2000 reagent. Approximately 30 h post-transfection the cells on coverslips were fixed in 4% formaldehyde for 15 min treated with 0.5% TritonX-100 in PBS for 10min and blocked with 3% BSA in PBS for 30 min. The cells were then incubated with a rabbit anti-Flag and/or mouse anti-HA antibodies followed by an Alexa Fluor 495-labelled and/or 594-labelled secondary antibody. Nuclei were counterstained with Hoechst33258. The cells were then examined using a confocal laser scanning microscope (Leica TCS NT).

## Additional Information

**How to cite this article**: Han, R. *et al.* Trim69 regulates zebrafish brain development by ap-1 pathway. *Sci. Rep.* **6**, 24034; doi: 10.1038/srep24034 (2016).

## Supplementary Material

Supplementary Information
