# Supplementary material for: Trim69 regulates zebrafish brain development by ap-1 pathway
Source: Sci Rep. 2016 Apr 6;6:24034. doi: 10.1038/srep24034 (PMC4822136; doi:10.1038/srep24034)
Supplement: Supplementary Information [file srep24034-s1.pdf]

Trim69 regulates zebrafish brain development by ap-1 pathway

Ruiqin Han [1#], Renxian Wang[1#], Qing Zhao[1], Yongqing Han[1], Shudong Zong[2], Shiying Miao[1], Wei Song[1*], Linfang Wang[1*]

[1]National Laboratory of Medical Molecular Biology, Institute of Basic Medical Sciences, Chinese Academy of Medical Sciences and Peking Union Medical College, Beijing, 100005, China;

[2]National Health and Family Planning Commission of the People’s Republic of China, WHO Collaboration Center of Human Reproduction, Beijing 100081, China.

[#]These authors contributed equally to this work. *Correspondence and requests for materials should be addressed to Wei Song (email: roy_sw0925@sina.com) and Linfang Wang (email: lfwangz@yahoo.com).

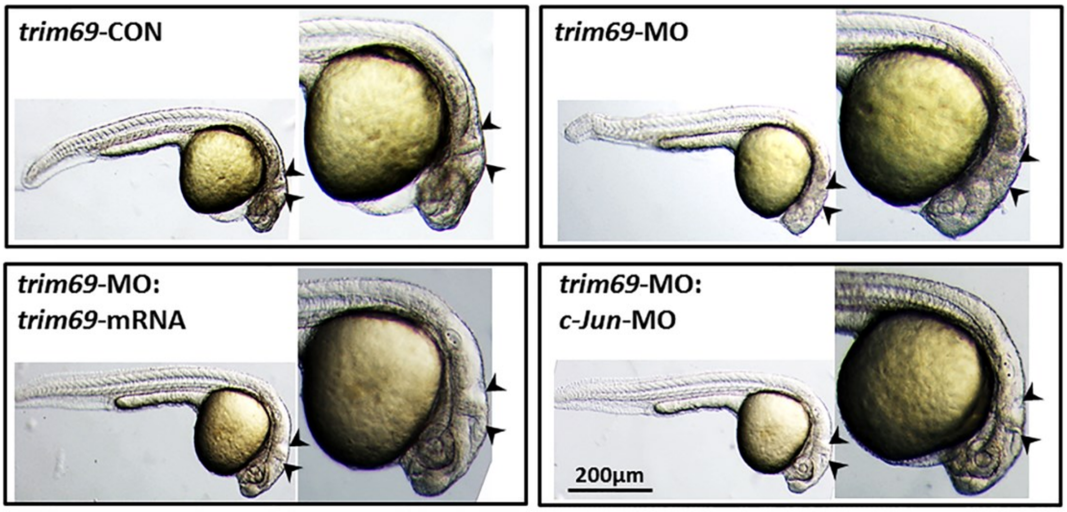

Figure s1. *Trim69* knockdown induces deformed brain.

trim69-CON: control group; *trim69*-MO: *trim69* knocking down; *trim69*-MO:*trim69*-mRNA: co-injection with *trim69*-MO and human *trim69* mRNA; *trim69*-MO:*c-Jun*-MO: co-injection with *trim69*-MO and *c-Jun*-MO; black arrow indicates mid-hind brain boundary(MHB); scale bar: 200 μm;

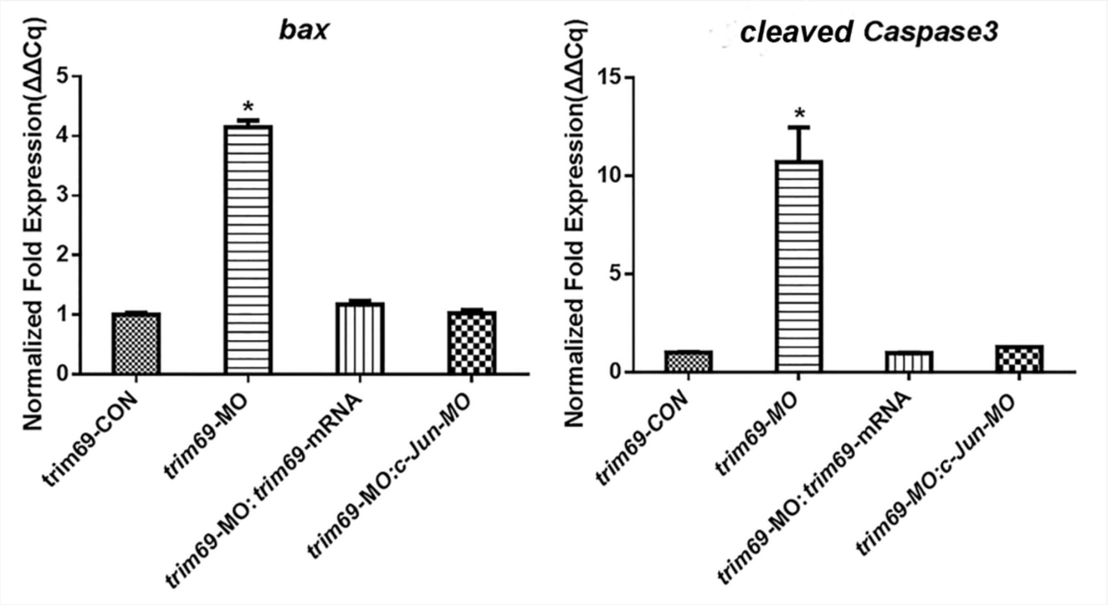

Figure s2. Expression of *bax* and *cleaved caspase3* detected by qPCR.

trim69-CON: control group; *trim69*-MO: *trim69* knocking down; *trim69*-MO:*trim69*-mRNA: co-injection with *trim69*-MO and human *trim69* mRNA; *trim69*-MO:*c-Jun*-MO: co-injection with *trim69*-MO and *c-Jun*-MO; Data were measured in triplicate and statistically analyzed by unpaired t test, p<0.01; values and bars represent the mean and standard deviation, respectively.

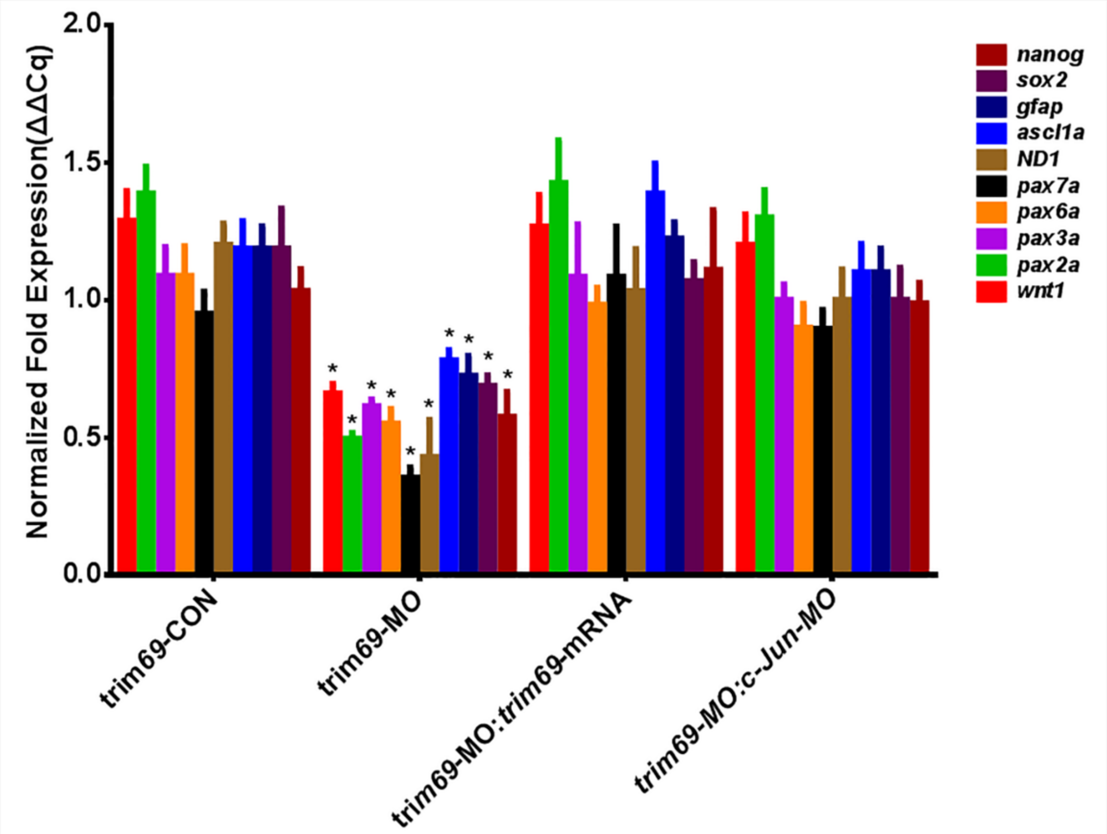

Figure s3. Expression of neuronal differentiation and stem cell markers detected by qPCR. *trim69*-CON: control group; *trim69*-MO: *trim69* knocking down; *trim69*-MO:*trim69*-mRNA: co-injection with *trim69*-MO and human *trim69* mRNA; *trim69*-MO:*c-Jun*-MO: co-injection with *trim69*-MO and *c-Jun*-MO; Data were measured in triplicate and statistically analyzed by unpaired t test, $p<0.01$; values and bars represent the mean and standard deviation, respectively.

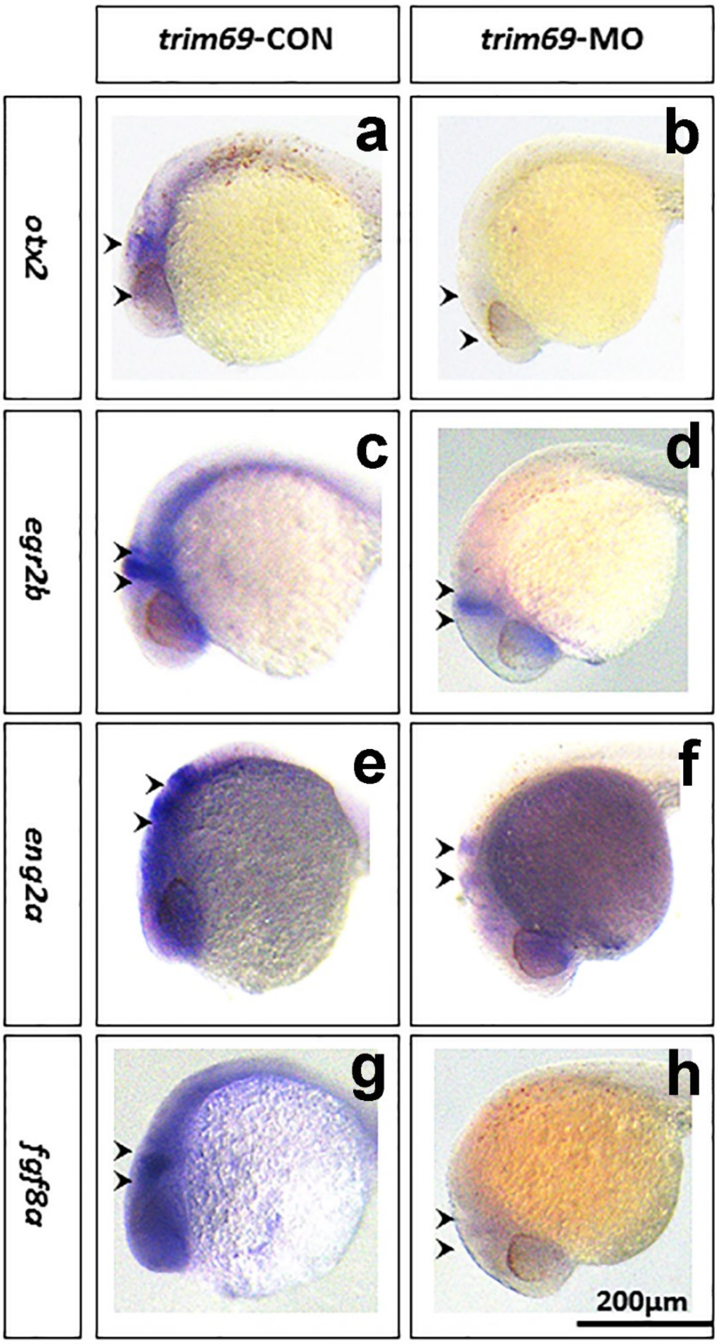

Figure s4. Expression of brain region specific markers detected by *in situ* hybridization.

Detection of the expression of *otx2* (marker of forebrain and MHB), *eng2a* (marker for MHB and hindbrain), *egr2b* (marker for MHB and hindbrain), and *fgf8a* (marker for forebrain and MHB) after loss of *trim69* by *in situ* hybridization. *trim69*-CON: control group; *trim69*-MO: *trim69* knocking down; black arrow indicates positive signals; scale bar: 200 μ m;
